# Potassium fertilization arrests malate accumulation and alters soluble sugar metabolism in apple fruit

**DOI:** 10.1242/bio.024745

**Published:** 2018-11-07

**Authors:** Wen Zhang, Xian Zhang, Yufei Wang, Nishang Zhang, Yanping Guo, Xiaolin Ren, Zhengyang Zhao

**Affiliations:** 1 College of Horticulture, Northwest A&F University, Yangling 712100, Shaanxi, China; 2 Key Laboratory of Horticulture Plant Biology and Germplasm Innovation in Northwest China, Ministry of Agriculture, Northwest A&F University, Yangling 712100, Shaanxi, China; 3 Shaanxi Engineering Research Center for Apple, Northwest A&F University, Yangling 712100, Shaanxi, China

**Keywords:** Apple fruit, Calcium, Malate, Potassium, Soluble sugar

## Abstract

Effects of different potassium (K) levels, which were K0 (no fertilizer), K1 (71.5 g KCl plant^−1^ year^−1^), K2 (286.7 g KCl plant^−1^ year^−1^), and K3 (434 g KCl plant^−1^ year^−1^), were evaluated based on sugar and organic acid metabolism levels from 70–126 days after bloom (DAB) in the developing fruit of potted five-year-old apple (*Malus domestica*, Borkh.) trees. The results indicate that K fertilization promoted greater fruit mass, higher Ca^2+^ and soluble solid levels, and lower titratable acid levels, as well as increased pH values at harvest. With the application of different levels of K fertilizer, fructose, sorbitol, glucose and sucrose accumulation rates significantly changed during fruit development. Fruit in the K2 group had higher fructose, sucrose and glucose levels than those in other treatment groups at 126 DAB. These changes in soluble sugar are related to the activity of metabolic enzymes. Sucrose synthase (SS) and sorbitol dehydrogenase (SDH) activity in the K2 treated fruit was significantly higher than those in other treatment groups from 70–126 DAB. Malate levels in K-supplemented fruit were notably lower than those in non K-supplemented fruit, and K3 treated fruit had the lowest malate levels during fruit development. Cytosolic malic enzyme (ME) and phosphoenolpyruvate carboxykinase (PEPCK) activity significantly increased in fruit under the K2 treatment during 112–126 DAB and 98–126 DAB, respectively. In addition, Ca^2+^ concentration increased with increasing K fertilization levels, which promoted a maximum of 11.72 mg g^−1^ dry weight in apple fruit. These results show that K levels can alter soluble sugar and malate levels due to the interaction between sugars and acid-metabolic enzymes in fruit.

## Abstract (precis)

**Summary:** Optimized potassium levels not only reduce malate levels but also improve fructose and sucrose accumulation in apple fruit.

## INTRODUCTION

Apples (*Malus domestica*, Borkh.), belonging to the Rosaceae family, are one of the most important cultivated fruit crops grown worldwide. China is the greatest apple producing country in the world with yields accounting for slightly more than 49% of total world production (FAO, 2012). Improved fruit quality is important for increasingly successful apple cultivation. The metabolic mechanisms of sugars and organic acids in fruit play important roles in fruit yield and quality, and determine fruit organoleptic characteristics.

In apples, both sorbitol and sucrose are first synthesized in leaves, then translocated to and used in fruit, in which the carbohydrate level consists of about 70% sorbitol and 30% sucrose (Klages et al., 2001). Sorbitol is taken up into the cytosol and is then converted to fructose by sorbitol dehydrogenase (SDH, EC1.1.1.14). Sorbitol can also be converted to fructose and glucose by neutral invertase (NINV, EC 3.2.1.26), or be converted to fructose and UDP-glucose (UDPG) by sucrose synthase (SUSY, EC 2.4.1.13) (Li et al., 2012). Most of the malic acid in apple fruit is found in the vacuole, which contains 85–90% of the total malic acid levels (Yamaki, 1984). Malic acid accumulation in apple fruit is well known to be under the tight control of several key enzymes, such as phosphoenolpyruvate carboxylase (PEPC), phosphoenolpyruvate carboxykinase (PEPCK) and malate dehydrogenase (MDH). In the metabolic pathways of the dicarboxylate malate, the initial formation of malate is carboxylation of phosphoenolpyruvate (PEP) in the cytosol, then the degradation of decarboxylation of malate and oxaloacetate (OAA), and finally those that allow conversion between tri- and dicarboxylates. These require fixation of CO_2_ on a carbon skeleton derived from hexose catabolism, which is achieved by the carboxylation of PEP, catalyzed by the phosphoenolpyruvate carboxylase (PEPC). This reaction takes place in the cytosol, since PEP is an intermediate of the glycolysis pathway, and produces OAA, which can then be reduced to malate by the cytosolic NAD-dependent malate dehydrogenase (NAD-cytMDH) (Etienne et al., 2013). MDH and PEPC participate in malic acid synthesis, while the action of the cytosolic NADP dependent malic enzyme (ME) results in malic acid degradation. Phosphoenolpyruvate carboxykinase (PEPCK) is also implicated in malic acid dissimilation, as it may be converted to sugar via gluconeogenesis (Walker et al., 2002). Meanwhile, not only malic acid levels, but also fructose and sucrose levels increased in over-expressing MDH apple plants (Yao et al., 2011). There are close relationships between sugar accumulation and acid metabolism during fruit quality formation (Yao et al., 2011).

Potassium (K) is an essential plant mineral element for plant development. Potassium cations (K^+^) are first absorbed by the apex cells of roots and then transported through the xylem to leaves and fruit (Brown and Cartwright, 1953). There are high- and low-affinity K^+^ uptake systems, which include carriers, channels or active pumps on the plasma membrane and tonoplast of plant cells (Santa-María et al., 1997). Potassium is transported across the tonoplast by a specific transporter protein and accumulates in the vacuole (Fontes et al., 2011). Under potassium starvation or salt stress, K^+^ is released from the vacuole providing K^+^ homeostasis in the cytoplasm, as well as for the cellular maintenance of turgor, enzyme activation and membrane potential (Fontes et al., 2011; Nieves-Cordones et al., 2014). Adequate K nutrition greatly influences the synthesis of sucrose and starch in plants such as apple (Mosa et al., 2015), muskmelon (Lester et al., 2010), tomato (Almeselmani et al., 2010) and strawberry (Ahmad et al., 2014). However, K levels have different effects on organic acid metabolism depending on the plant species (Etienne et al., 2014; Flores et al., 2015; Niu et al., 2016).

Most research has focused on the relationship between K levels and the parameters of hardness, soluble sugar or titratable acid. Also, only a few studies directly address the importance of K mechanisms for improving fruit quality at the biochemical level (Lin et al., 2004; Kanai et al., 2007; Lizarazo et al., 2013). In this study, the importance of different K levels on ‘Gala’ apple fruit were evaluated based on fruit growth patterns, sugar and acid levels, and sugar-metabolic and malate-metabolic enzymatic assays.

## RESULTS

### Effect of different levels of K fertilization on fruit characteristics during fruit development

**Fig. 1.**
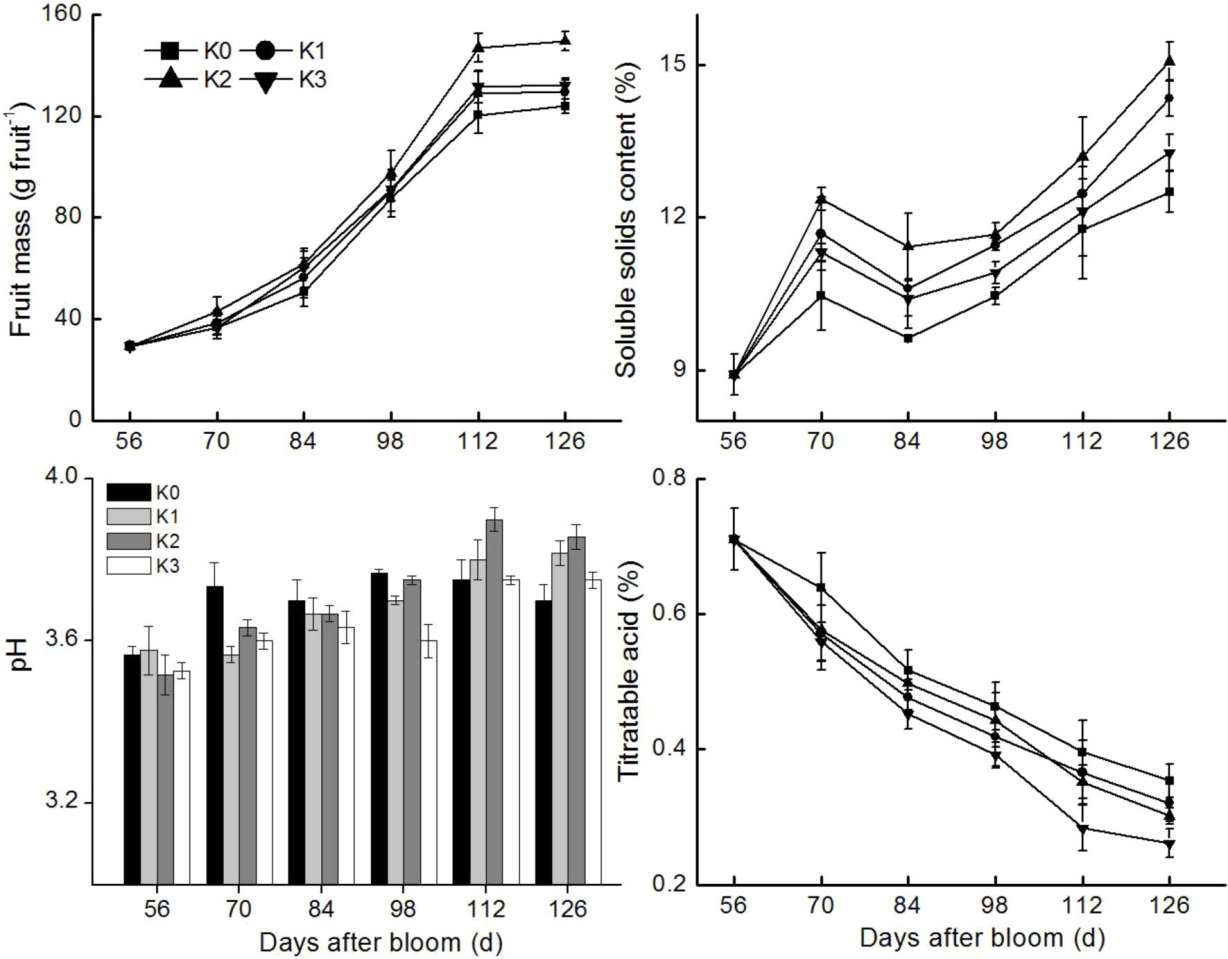
**Influence of different K levels on fruit mass, soluble solids levels, pH and titratable acid during apple fruit development (*n*=5).**

A profile of apple fruit growth was established by measuring the changes in fruit mass, pH, soluble solid levels and titratable acid under different K-level conditions (Fig. 1). Fruit mass followed a fast-slow rule during the time period from 56–126 days after bloom (DAB); the fruit rapidly expanded until 112 DAB, and then a slower growth period was observed from 112–126 DAB as the fruit matured. Fresh mass of K-treated fruit was higher than that of non-K-treated fruit at the fruit maturity stage (112–126 DAB). Moreover, the fresh mass of K2-treated fruit was higher than that of non-K-treated fruit from 56–126 DAB. Fruit production under the K2 treatment was the highest at 126 DAB (Table 1). As shown in Fig. 1, pH values of K0 fruit were first elevated and then gently fluctuated. The pH values of K0-treated fruit were higher than those measured in other K-treatment groups at 70 DAB and K2-treated fruit had the highest pH values during 112–126 DAB.

**Table 1.**
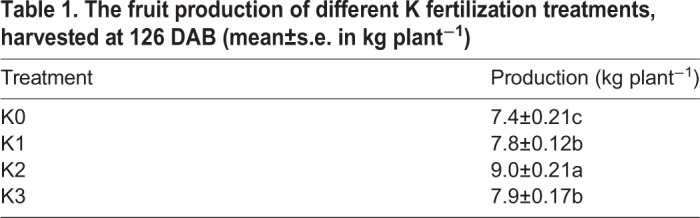
**The fruit production of different K fertilization treatments, harvested at 126 DAB (mean±s.e. in kg plant^−1^)**

Soluble solid levels of fruit increased during development of apple fruit (Fig. 1). When comparisons were made between each group, soluble solid levels of fruit were the highest in the K2 treatment at 126 DAB. Soluble solid levels of fruit in the K0 treatment were lower than those of fruit in any other K treatment from 56–126 DAB. Titratable acid concentration in apple fruit decreased with fruit growth and development and followed the opposite trend of soluble solid levels from 56–126 DAB (Fig. 1). Titratable acid levels of fruit assayed in the different K treatments were significantly lower than those of K0-treated fruit, with K3-treated fruit having the lowest. In order to investigate the effect of different levels of K in fertilizer on the taste of apple fruit, sugar-acid ratios were evaluated (Table 2). Sugar-acid ratio was determined by dividing the amount of malate by the sum of fructose, sorbitol, glucose and sucrose. The sugar-acid ratio of fruit under all K treatments was higher than that found in the non-K-treatment groups at 126 DAB. The sugar-acid ratio in K2-treated fruit was higher than any other treatment.

**Table 2.**
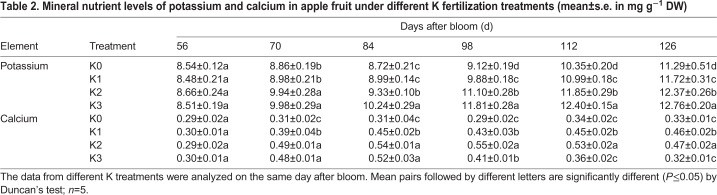
**Mineral nutrient levels of potassium and calcium in apple fruit under different K fertilization treatments (mean±s.e. in mg g^−1^ DW)**

### Effect of different levels of K fertilization on calcium concentration during fruit development

During fruit development, K levels in fruit were on the rise and were significantly elevated with increasing K levels at each time point from 56–126 DAB (Table 1). There was no significant difference between K0-treated fruit and K1-treated fruit in K levels during 56–84 DAB, and a rising trend was observed after 84 DAB. Meanwhile, K levels in K2- and K3-treated fruit showed significant differences from K0-treated fruit from 56–126 DAB. Among all the treatments, fruit under the K3 treatment had the highest K levels from 56–126 DAB.

Calcium (Ca) concentration in K0-treated fruit gently fluctuated from 56–126 DAB, and was different from that of all K-treated fruit (Table 2). Calcium levels of all K-treated fruit significantly increased compared to K0-treated fruit and the K2 treatment promoted the highest calcium levels from 56–126 DAB. After 98 DAB [K level was 11.81±0.28 mg g^−1^ dry weight (DW)], the Ca levels of fruit in the K3 treatment gradually decreased. Calcium levels of K2-treated fruit were also reduced after 112 DAB (K level was 11.72±0.31 mg g^−1^ DW). These indicate that K and Ca in fruit accumulated with increases in K applications, but Ca in fruit accumulated within limits. When K accumulation in fruit was greater than 11.72 mg g^−1^ DW, the Ca accumulation in fruit showed a downward trend.

### Metabolic profiling during fruit development

**Fig. 2.**
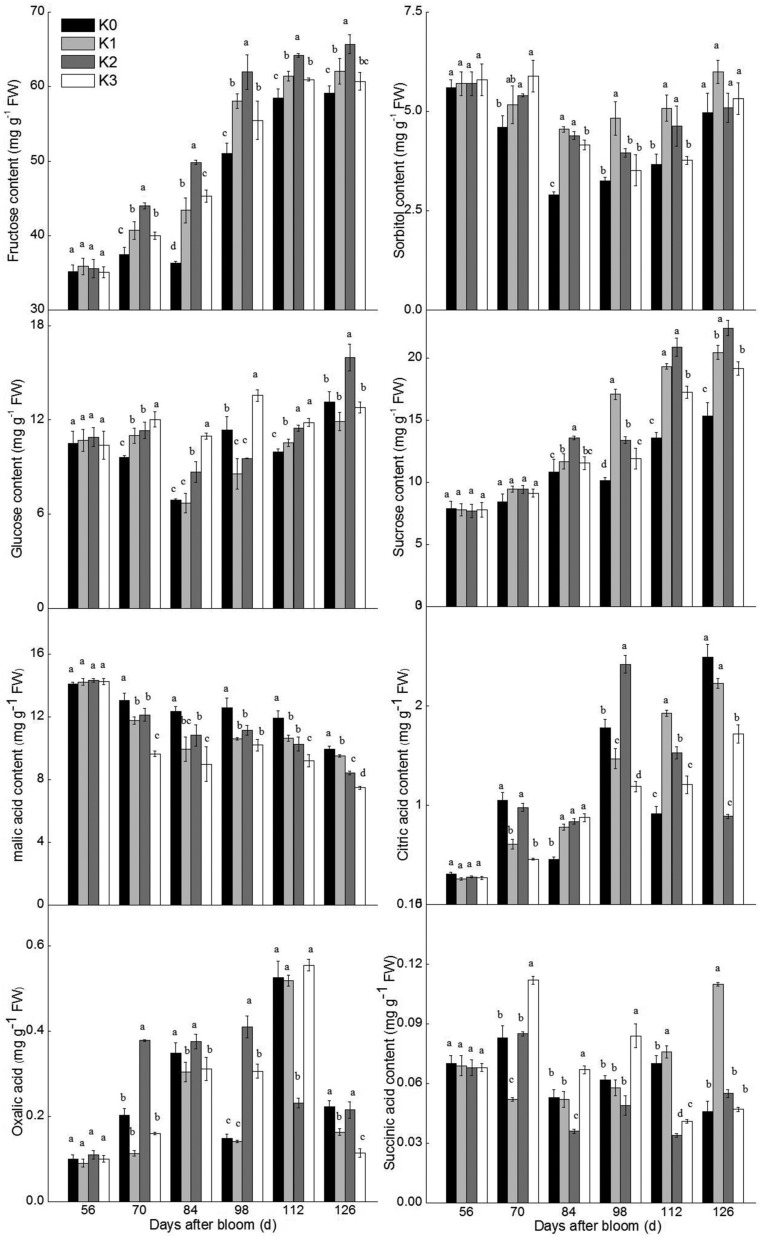
**Soluble sugar and organic acid levels with different K fertilization during fruit development.** The data of different K treatments were analyzed the same day after bloom. Different letters represent significant differences at *P*≤0.05 by Duncan's multiple range test; *n*=3. Results are presented as mean±s.e. in mg g^−1^ FW.

As shown in Fig. 2, fructose, glucose and sucrose levels increased and reached peak levels at 126 DAB. Sorbitol levels first decreased from 56–84 DAB and gradually increased thereafter. Fructose levels had the greatest accumulation among all soluble sugars examined, with fructose showing the highest accumulation in the K2-treated fruit from 56–126 DAB. Moreover, fructose levels of K3-treated fruit were similar to those of K1-treated fruit, and were higher than those of K0-treated fruit during fruit development. A significant rise in sucrose accumulation in all K-treated fruit was observed by comparing fruit in the K0 treatment to fruit in all other treatment groups from 98–126 DAB. The variation trend of glucose levels in fruits was similar to fructose during apple development. There were no obvious differences in glucose and sorbitol levels among all treatments during fruit development. Thus, the K2 treatment efficiently improved the accumulation of fructose and sucrose in fruit, but had little effect on glucose and sorbitol levels. The K1 and K3 treatments also promoted fructose and sucrose levels in fruit, but not as much as the K2 treatment did.

In addition to soluble sugars, malate is also a main soluble component in apple fruit (Etienne et al., 2013). During fruit growth, decreasing levels of malic acid in fruit were observed from 56–126 DAB (Fig. 2). Malic acid levels of fruit in the three K-treatment groups were significantly lower than those in the control treatment. From 56–126 DAB, the malic acid level of K3-treated fruit was the lowest among all the treatments. Other organic acids (oxalic acid, citric acid and succinic acid) among three K-treated fruit showed no significant differences from those in the control treatment.

### Activities of key enzymes in sugar and malate metabolism under different K fertilization conditions

**Fig. 3.**
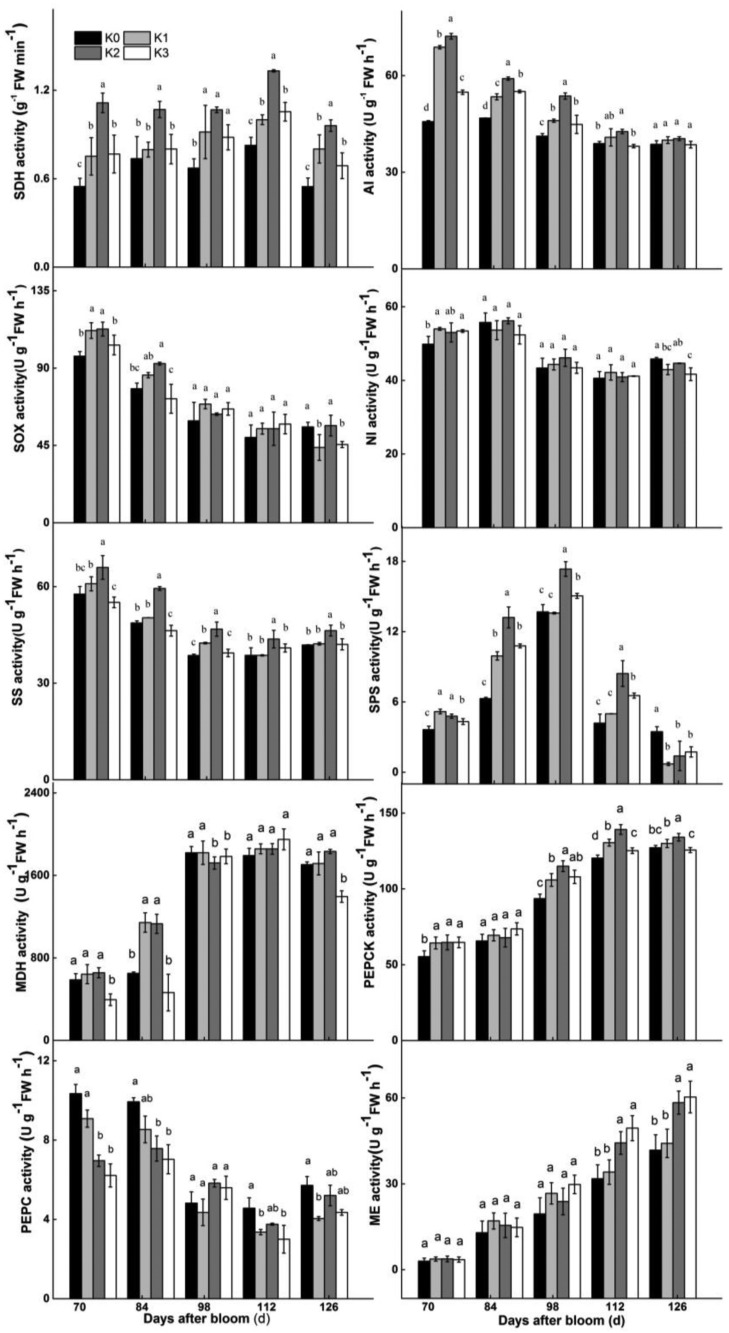
**Changes of sugar- and malate-metabolic enzyme activities during fruit development under different K treatment conditions.** SDH, sorbitol dehydrogenase; SOX, sorbitol oxidase; AI, acid invertase; NI, neutral invertase; SS, sucrose synthase; SPS, sucrose phosphate synthase. PEPC, phosphoenolpyruvate carboxylase; MDH, malic dehydrogenase; ME, malic enzyme; PEPCK, phosphoenolpyruvate carboxykinase. The data of different K treatments were analyzed on the same day after bloom. Different letters represent significant differences at *P*≤0.05 by Duncan's multiple range test; *n*=3.

To further understand sugar and malate metabolism, the activities of relevant enzymes were analyzed (Fig. 3). SDH activity in K2-treated fruit was higher than in fruit assigned to other treatments from 56–112 DAB, and SDH activity in K0-treated fruit was the lowest. SOX activity in fruit decreased from 70 DAB, and there were no significant differences during fruit development across different K treatments. Neutral invertase (NI) activity levels across different K-treated fruit also showed no differences from 56–126 DAB. Generally, acid invertase (AI) activity decreased during fruit development, but AI activity in K2-treated fruit only held this trend from 56–98 DAB and was otherwise notably higher than that of other treatments. SPS, the enzyme participating in sucrose re-synthesis, reached peak activity at 98 DAB and then declined rapidly. SPS activity in K2-treated fruit was the highest from 84–112 DAB and SPS activity of K0-treated fruit was the lowest from 56–112 DAB. Sucrose synthase (SS) activity in all treatment groups decreased, but SS activity in K2-treated fruit was notably higher than any other treatments during fruit growth and development. Therefore, the K2 treatment markedly promoted SDH and SS activity from 56–126 DAB, and only improved AI and SPS activity from 56–98 DAB and 84–112 DAB, respectively.

The key enzymes of malate metabolism are also reported in Fig. 3. PEPC and MDH, which regulate malate synthesis, showed an inverse trend during fruit growth. PEPC activity first showed a significant reduction from 56–112 DAB and then increased toward maturity, while MDH activity was elevated from 56–98 DAB and declined at 125 DAB. In addition, both PEPC and MDH activity in K3-treated fruit was lower than that in other treatments from 56–84 DAB and at 126 DAB. PEPCK and ME, which are involved in malate decomposition, showed a rising trend during fruit development. PEPCK activity in K3-treated fruit was significantly lower than other treatments from 112-126 DAB. However, ME activity in both K2- and K3-treated fruit was notably higher from 112–126 DAB. Thus, none of the enzymatic activity involved in malate metabolism was influenced by K treatments throughout all stages of fruit development.

## DISCUSSION

### Very high levels of K fertilization are not the best way to improve fruit characteristics

Potassium is an essential plant mineral nutrient for improving fruit quality (Lester et al., 2006; Kanai et al., 2007; Lizarazo et al., 2013). Higher K levels increase fruit weight, size, firmness or soluble sugar levels in citrus (Lin et al., 2006), apple (Nava et al., 2008) and melon (Lin et al., 2004). It is conceivable that K strengthens photosynthetic product transport to fruits (Berüter and Studer, 1997). In our study, soluble solid levels significantly increased with fruit development (Fig. 1). The soluble solid is from the photosynthetic product, hence K improved the photosynthetic product transport from leaves to fruit in apples. This phenomenon was also found in papaya (Ghosh and Tarai, 2007) and banana (Isayenkov et al., 2010).

In addition to higher soluble sugar levels, fruit acidity is another important component of fruit organoleptic quality (Etienne et al., 2013). It has been reported that in citrus titratable acid levels continuously decline with fruit ripening (Cercos et al., 2006), and fruit acidity is negatively correlated with supplemental K fertilization in banana (Kumar and Kumar, 2007) and pineapple (Spironello et al., 2004). In the present study, titratable acid levels showed a trend of rapid decline from 56–126 DAB across all treatments (Fig. 1). K3-treated fruit containing the highest K levels had the lowest titratable acid levels at 126 DAB. Furthermore, different K fertilization levels altered different sugar-acid ratios in fruit. The sugar-acid ratios of fruit assigned to the K2 treatment were the highest (Table 3). Thus, the K2 treatment provided the most beneficial environment for the accumulation of soluble solid levels, increased fruit mass, fruit production, pH value and sugar-acid ratio in fruit (Fig. 1; Tables 1 and 3). The K3 treatment decreased titratable acid levels in fruit more than any other treatment.

**Table 3.**
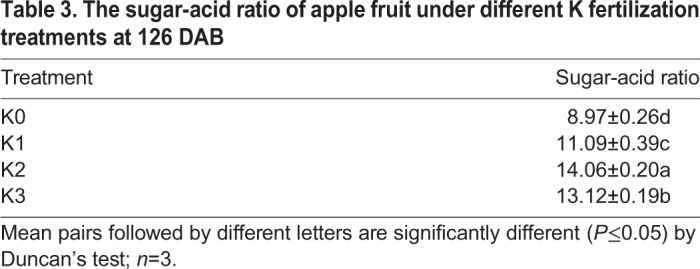
**The sugar-acid ratio of apple fruit under different K fertilization treatments at 126 DAB**

### Optimized K application promoted higher Ca accumulation but Ca uptake was inhibited by excessive K

Potassium is not structurally bound to organic tissue but rather is used as a water-soluble salt (Palviainen et al., 2004). Release of Ca is dependent on the microbial decomposition of its structural compounds, so Ca leaching release was less than that exhibited by K (Ukonmaanaho and Starr, 2001). Calcium has been shown to ameliorate the negative effects caused by salinity (Adams and Shin, 2014). Potassium accumulation in the soil affects Ca availability in the surface and subsurface layers, mainly in areas receiving high K rates (Nachtigall et al., 2007). As shown in Table 2, all K applications enhanced K accumulation in fruit from 56–126 DAB. The increasing quantity of K in K0- and K1-treated fruit was first noted after 112 DAB and then leveled off. However, the increasing quantity of K levels in K2- and K3-treated fruit began to slow down at 98 DAB, after which the rate of K acquisition was gradually even slower. Calcium levels in the K0- and K1-treated fruit gradually increased from 56–112 DAB and then leveled off. Interestingly, the trend of Ca accumulation in K0- and K1-treated fruit was similar to that of K acquisition rates in fruit.

A decreasing trend of Ca levels was observed in both K2- and K3-treated fruit, which was different from the trend in K0- and K1-treated fruit. Johansen et al. (1968) and Nachtigall et al. (2007) found that increasing K levels inhibited Ca absorption. Calcium levels in K2-treated fruit first increased from 56–84 DAB, evened out from 84–112 DAB and then decreased at 126 DAB. Calcium levels in K3-treated fruit first increased from 56–84 DAB and then Ca levels decreased after 84 DAB. Calcium levels of fruit in the K3 treatment began to reduce at 84 DAB, which was earlier than that of the K2 treatment (112 DAB). There is a reason for these changes of K and Ca levels in fruit. Possibly, there is an upper limit for K concentration that exists in the tissues (Leigh, 2001), and since K was continually applied in this study, the change in rate of K levels in the K2 and K3 treatments continued to slowly and gradually increase after 98 DAB. Meanwhile, Ca levels of K2- and K3-treated fruit did not increase after 98 DAB. The Ca levels of fruit in the K3 treatment were reduced after 98 DAB, while Ca levels of fruit in the K2 treatment reduced at 126 DAB. Potassium levels of fruit in the K2 treatment at 126 DAB and that of K3 treatment at 98 DAB were 11.72±0.31 and 11.81±0.28 mg g^−1^ DW, respectively. In this study, 11.72±0.31 mg g^−1^ DW of K in fruit was the optimal concentration that most promoted Ca levels in fruit.

### Effect of different K levels on soluble sugar metabolism

In the Rosaceae family, sorbitol and sucrose are the main photosynthetic products transported (Shangguan et al., 2014). When sorbitol and sucrose are transported to fruit, a variety of sugars are synthesized (Li et al., 2012). As shown in Fig. 2, fructose accumulated in much higher levels than glucose, sucrose or sorbitol during fruit development. The K2 treatment resulted in the increase of sucrose, glucose and fructose levels, but there was no significant difference in sorbitol levels when compared to fruit in the K0 treatment at 126 DAB. These changes in sucrose, glucose and fructose levels were similar to those of netted muskmelon (Lester et al., 2006). In addition, highest K levels of the treatments, which were 434 g KCl plant^−1^ year^−1^, did not improve fruit sucrose, glucose and fructose accumulation during fruit development from 56 to 126 DAB.

To better understand the change of soluble sugar levels under different K applications, enzymatic activity in soluble sugar metabolism were analyzed during fruit development (Figs 2 and 3). Rather than K3-treated fruit, K2-treated fruit had the highest SDH activity during fruit development. This trend in SDH enzyme activity coincided with that of fructose accumulation during fruit development. Different K treatments had a slight influence on SOX activities. SPS and SDH activity play an important role in sucrose synthesis, especially SPS (Fan et al., 2009). In Figs 2 and 3, sucrose levels in apple were increasing with increasing SPS activity under the K2 treatment from 70–112 DAB. There were notable reductions of SS activity and gentle fluctuations of NI and AI activity during fruit development (Fig. 3). AI and SS are two other enzymes initially involved in sucrose breakdown (Shangguan et al., 2014). SS and AI activity showed a declining trend in all treatments during fruit development, but both SS and AI activity in K2-treated fruits was higher than in other treated fruits at 70–98 DAB. At the same time, the sucrose and fructose levels showed an increasing trend, but there was no significant difference among treatments. Further, NI activity was seemingly not influenced by different K treatments. Liu et al. (2012) also found that optimum K levels significantly enhanced AI and SS activity in the storage roots of sweet potato. Therefore, SDH, SS, AI and SPS activity all changed with K treatments and these four enzymes influenced sucrose and fructose levels.

Considering fructose levels exhibited the most accumulation among all the soluble sugars, SDH, AI and SS were further analyzed under different K applications. The K cation directly affects the structure of many enzymes, which are divided into two types (Cera, 2006). SDH activity of K2-treated fruit was higher than in other treatments, but SDH activity was not directly promoted by cations (Yamaguchi et al., 1994; Oura et al., 2000). Bantog et al. (2000) thought that a key regulatory step of SDH activity was at the transcriptional level. Therefore, SDH activity needs to be further studied at mRNA and protein levels under different K treatment conditions. AI activity of K2-treated fruit was higher than in other treatments from 70–98 DAB, but this activity is also not directly promoted by divalent cations and K cations in plants (Bhatia et al., 2012; Xu et al., 2015). SS activity is directly inhibited by K cations (Singal et al., 2009), but SS activity in K-treated fruit was not lower than in control fruit during fruit development (Fig. 3). Because K concentration had a close relationship with Ca levels in the development of different K-treated fruit (Table 1), K might regulate SS activity via Ca levels, which directly influences PEPP (phosphoenolpyruvate phosphatase), CDPK and so on (Fedosejevs et al., 2016). Generally, K did not directly affect SDH, AI and SS activity in fruit.

### Effect of different K levels on malate metabolism

Malic acid is a dominant organic acid in apple fruit (Ulrich, 1970). As shown in Fig. 2, malic acid levels was much higher than citric acid, oxalic acid or succinic acid levels in fruit. Malic acid participates in the TCA cycle, glyoxylate cycle and the gluconeogenesis pathway during fruit development (Etienne et al., 2013). PEPC activity, which participates in malate synthesis, decreased in developing fruit (Fig. 3). PEPCK and ME activity were also reduced (Fig. 3). As a result, malic acid levels gradually decreased from 73–126 DAB (Fig. 2), in agreement with Yao et al. (2009) and Ruffer et al. (1984). However, Osorio et al. (2013) demonstrated that malate levels had a remarkably negative correlation with PEPCK activity in tomato. Thus, malic acid levels might not only be influenced by malate-related enzyme activity but may also be affected by other factors.

With the increase of level of K in the soil, the citric acid and oxalic acid levels in fruit significantly increased, nevertheless, the malic acid level were significantly reduced. On the other hand, the level of succinic acid in fruit showed no regular change during fruit development (Fig. 2). Coincidentally, Lobit et al. (2006) also found that increasing K levels in cells were accompanied by a decrease in the accumulation of malic acid. Moreover, K levels in fruits were negatively correlated with acid levels in low acid apple cultivars (Berüter, 2004). Potassium is an active ion involved in ME activity (Suelter, 1970). However, after 112 DAB, ME activity in K3-treated fruit was significantly higher than that in the control fruit (Fig. 3). MDH activity in K1- and K2-treated fruit was notably higher than in the control treatment from 70–84 DAB, but K3-treated in fruit showed no regular change. PEPC activity is stimulated by both elevated K^+^ and Ca^2+^ in plants (Balkos et al., 2010; Park et al., 2015), however, in our study, different K-treated fruit did not have higher PEPC activity than fruit in the control treatment. PEPC activity in K2-treated fruit was higher than that in K1- and K3-treated fruit, but it was lower than that in K0-treated fruit (Fig. 3). Thus, the enzymes of malate metabolism were not specifically and directly influenced by both ions of K+ or Ca^2+^ in plants.

**Fig. 4.**
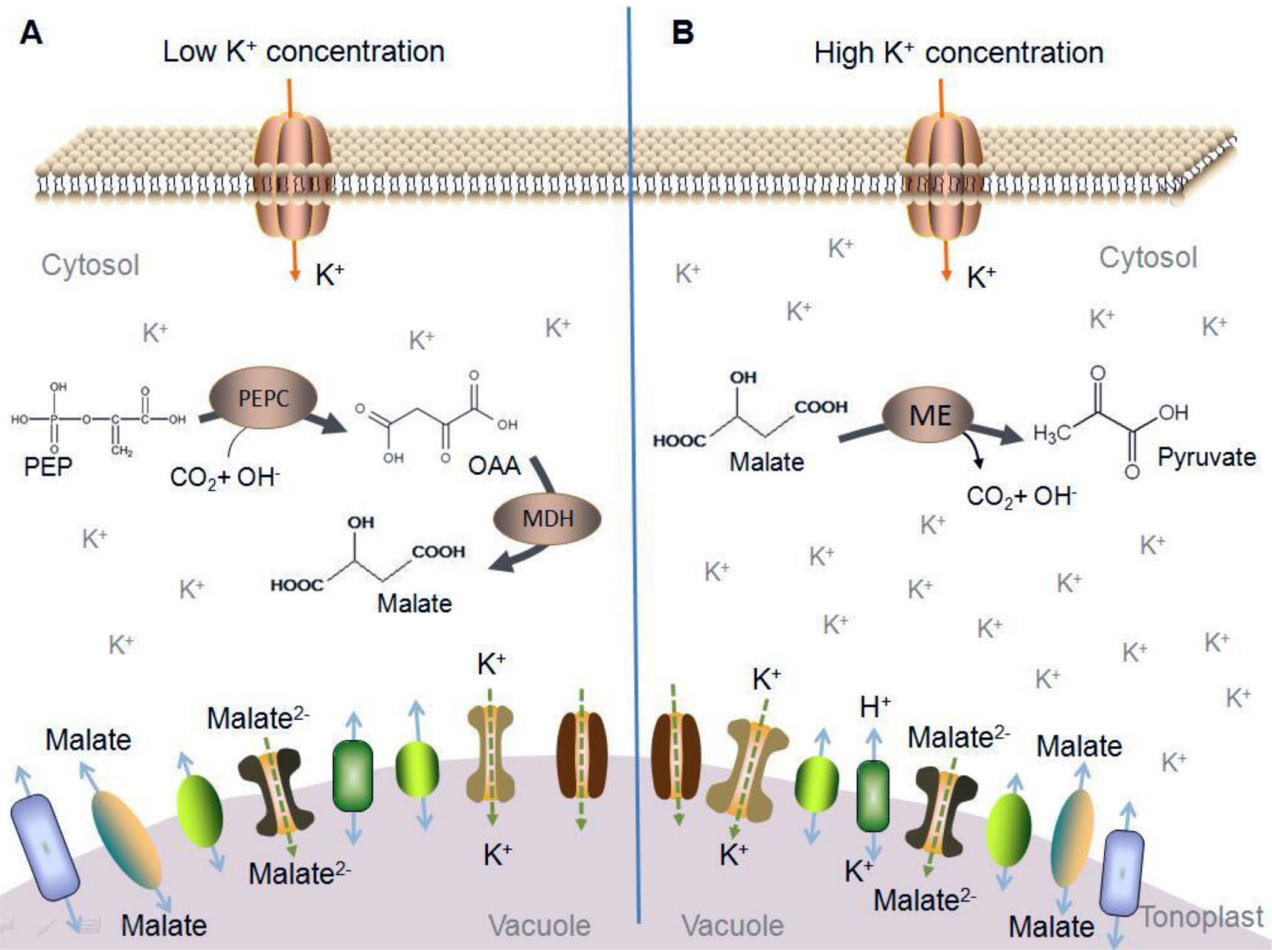
**The model of malate metabolism in fruit cells under different K level conditions.** Different colored shapes located on the tonoplast and plasma membrane represent different transport proteins. The brown ovals in the cytosol indicate enzymes involved in malate metabolism. (A) Malate metabolism in fruit under low K level condition; (B) malate metabolism in fruit under high K level condition.

Malate accumulation in fruit was not determined by changes in metabolism but by the conditions of its transport into vacuoles (Lobit et al., 2006). When K levels were increased in the fruit cell, most of K^+^ was transported to the vacuole (Etinne et al., 2013). In this work, a speculative model of malate metabolism under different K level conditions was developed (Fig. 4). When K is transported into fruit cells at a low concentration, facilitated diffusion through a vacuolar cation channel is the most likely mechanism (Isayenkov et al., 2010). When the diffusion of anions through a special ion channel occurs, malate accumulates in the vacuole, which leads to alkalization that promotes malate synthesis (Gout et al., 1993; Etinne et al., 2013; Lobit et al., 2006). During malate synthesis, protons are released into the cytosol (Etinne et al., 2013). Thus, pH values of K1-, K2- and K3-treated fruit were lower than those found in the control group from 56–98 DAB (Fig. 1). When a high K concentration is found in fruit cells, active transport is required from a K^+^/H^+^ anti-port, which mediates an electroneutral exchange (Etinne et al., 2013). Transporting K+ react with organic acid in fruit and salt is produced, so levels of organic acids are lower. Meanwhile, potassium absorption increases the release of malic acid, favoring its metabolism in the cytoplasm (Fontes et al., 2011). This might be one of the reasons that higher K levels in fruit resulted in the lower malate levels observed from 98–126 DAB (Fig. 2). During malate degradation via malic enzymes, OH^−^ ions are released, which leads to an increase in pH value (Fig. 1). Therefore, malate and malic enzymes play a fundamental role not only in fruit taste, but also in the regulation of cytosolic pH (Martinoia et al., 2007).

In conclusion, optimized K fertilization improved fruit quality at harvest. On the one hand, K indirectly improved AI, SS and SDH activity, which lead to an increase in soluble sugar levels during fruit development. On the other hand, K reduced malate levels in fruit and altered malic enzymatic activity through a pH homeostasis mechanism.

## MATERIALS AND METHODS

### Plant materials

In this study, fruit were harvested from six-year-old ‘Gala’ apple (*Malus domestica* Borkh.) trees, which were grafted onto M26 (*Malus robusta* Rehd.) self-rooted rootstock grown in mixed soil (20 kg per pot, volume ratio of field topsoil to organic manure was 2:1, pH 7.2) in plastic pots (50 cm diameter, 45 cm tall). The trees were kept in an experimental orchard located at Northwest A&F University (34°20′ N, 108°24′ E). In order to avoid roots grown through the bottom of the pots in the field, all containers were covered with plastic film and buried. The fruit trees were thinned and approximately 60 fruit were on each tree. Before K treatment, basic soil fertilization consisted 16.6±0.54 g of organic matter kg^−1^, with a pH of 7.2±0.02, 46.5±2.8 mg kg^−1^ of available nitrogen, 17.3±1.7 mg kg^−^^1^ of available phosphorus, and 68.2±2.1 mg kg^−^^1^ of available potassium.

Trees were planted in a randomized design. Thirty-two apple trees of the same age and developmental stage were divided into four groups; each group included eight repeats (each tree was considered one replication). Trees were fertilized with a KCl reagent and the four treatments were as follows: K0 (no fertilizer), K1 (71.5 g KCl plant^−1^ year^−1^), K2 (286.7 g KCl plant^−1^ year^−1^) and K3 (434 g KCl plant^−1^ year^−1^). Soil fertilizer was applied within 20 cm of the tree trunk during the youngest developmental fruit period (fruit diameter up to 25 mm, used 20% of total K fertilizer), the fruit swelling period (fruit about 50–60% of final size, used 50% of total K fertilizer) and the fruit ripening period (fruit about 80–90% of final size, used 30% of total K fertilizer). Soil was fertilized 10 days before samples were collected. Samples were collected between 09:00–10:00 h from June 5 to August 14, 2015. The sample tissue was stored in 80°C for further analysis.

### Measurement of fruit characteristics and mineral levels

The following measurements were taken: fruit mass, pH, quality (total soluble solid, titratable acid), production and mineral concentrations. Fruit pH was analyzed with a measuring technique described by Xu et al. (2012). Total soluble solids and titratable acid concentration (%) were separately investigated with an electronic refractometer (PAL-1, Atago, Tokyo, Japan) and an acidity meter (GMK-855, G-won, Korea) in three measurements per biological replicate. In order to analyze fruit production, six fruit from one tree were harvested as one biological replicate at 126 DAB. There were five biological replicates.

Potassium levels were analyzed using the H_2_SO_4_/H_2_O_2_ digestion method and calcium was measured using the dry ash method (Benavides et al., 2001) in fruit tissue. Potassium levels were analyzed with flame emission spectroscopy (Z-2000, Hitachi High Technologies Corporation, Tokyo, Japan). Calcium levels were measured with atomic absorption spectrometry (Z-2000, Hitachi High Technologies Corporation, Tokyo, Japan).

### Measurement of soluble sugars and organic acids

Soluble sugars and organic acids were extracted according to the following procedure. 1 g of frozen sample was mixed with 3 ml of 80% (v/v) methanol and was incubated at 80°C for 30 min. Soluble sugars were measured using HPLC (LC-10ATVP, Shimadzu, Kyoto, Japan) equipment with a refractive index detector (RID) (RID-10A), according to Suárez et al. (2012), with some modification. The separation was performed by using a NH_2_P-50 4E column (4.6×250 mm), with a particle size diameter of 5 μm and a guard column (Shodex Asahipak, Tokyo, Japan). All separations were kept at 30°C. The mobile phase was composed of 80% (v/v) acetonitrile, with a flow rate of 1 ml min^−1^. The injection volume of each sample was 25 μl.

Organic acids were measured using HPLC (LC-2010AHT, Shimadzu, Kyoto, Japan) equipment with an ultraviolet detector (SPD-10AVP), according to Suárez et al. (2008) with some modifications. The separation was performed by using a C18 column (250×4.6 mm), with a particle size diameter of 5 μm and a guard column (Agilent Technologies, USA). All separations were kept at 25°C. The detection wavelength was 210 nm. The mobile phase was composed of potassium dihydrogen phosphate adjusted to pH 2.8, with a flow rate of 0.7 ml min^−1^. The injection volume of each sample was 10 μl. All peaks of HPLC samples were assigned by comparing the retention times to those obtained from standards. Duplicate injections were performed and average peak areas were used for quantification. All solvents used were HPLC grade, and reference standards were obtained from Sigma-Aldrich for both sugars and organic acids. There were three biological replications.

### Enzyme assays

Sorbitol dehydrogenase (SDH, EC 1.1.1.14) was extracted, as described by Park et al. (2002) and Yamaguchi and Kanayama (1996) with some modifications. 0.5 g frozen sample was homogenized in 2 ml of 200 mM potassium phosphate buffer (pH 7.8) containing 1 mM EDTA, 10 mM sodium ascorbate, 1 mM dithiothreitol (DTT), 0.15% (v/v) Triton X-100, 1% (w/v) BSA and 2% (w/v) insoluble polyvinylpolypyrrolidone (PVPP). The homogenate was centrifuged at 13,000×***g*** for 15 min at 4°C. 1 ml of the supernatant was desalted with a Sephadex G25 PD-10 column (GE Healthcare, UK) equilibrated with 125 mM Tris-HCl (pH 9.6). SDH activity was measured in a 1 ml reaction mixture containing 500 mM Sorbitol, 1 mM NAD^+^ and desalted extract in 125 mM Tris-HCl (pH 9.0), and NADH production was determined at 340 nm.

For acid invertase (AI, EC 3.2.1.26), neutral invertase (NI, EC 3.2.1.26), sucrose synthase (SS, EC 2.4.1.13), sorbitol oxidase (SOX, EC 1.5.3.1) and sucrose phosphate synthase (SPS, EC 2.4.1.14) activity, the extraction was performed as described by Keller and Ludlow (1993). A total of 0.7 g of frozen tissue was ground in a chilled mortar with 3.5 ml of 50 mM Hepes-NaOH buffer (pH 7.5) containing 5 mM MgCl_2_, 1 mM EDTA, 2.5 mM DTT, 0.1% (w/v) BSA, 0.05% (v/v) Triton X-100 with 2% (w/v) Polyvinyl Pyrrolidone (PVP). The homogenate was centrifuged at 13,000×***g*** for 20 min at 4°C and was desalted immediately in a Sephadex G25 PD-10 column.

AI activity was assayed in a 500 μl incubation mixture containing 80 mM acetic acid/sodium acetate buffer (pH 4.5), 100 mM sucrose, and an aliquot of the desalted extract; NI: 80 mM Acetic acid/tripotassium phosphate buffer (pH 7.5), 100 mM sucrose, and desalted extract; SS: 80 mM MES (pH 5.5), 100 mM sucrose, 5 mM UDP, and desalted extract; SOX: citric acid/trisodium citrate buffer (pH 4.0), 200 mM sorbitol and desalted extract. These mixtures were all incubated at 30°C for 30 min, and the assay was stopped by adding 500μl of 3,5-dinitrosalicylic acid and boiling for 5 min. It was determined at 540 nm. The blank of each enzyme assay was denatured extract (Lowell et al., 1989). SPS activity was measured according to Zhou and Quebedeaux (2003). The enzyme activity was assayed for 30 min at 37°C in a 70 μl assay medium containing 50 mM Hepes-NaOH (pH 7.5), 15 mM MgCl_2_, 1 mM EDTA, 16 mM UDPG, 4 mM F6P, and 14 mM G6P. The reaction was determined at 480 nm.

To extract malate dehydrogenase (MDH, EC 1.1.1.37) and malic enzyme (ME, EC 1.1.1.40), 1 g of frozen tissue was added to 3 ml of pre-chilled of 200 mM Tris-HCl (pH 8.2) and ‘Buffer A’ containing 600 mM sucrose and 10 mM isoascorbic acid. The homogenate was centrifuged at 4000×***g*** for 20 min at 4°C. 2 ml of the supernatant was mixed with an equal volume of 200 mM Tris-HCL (pH 8.2) ‘Buffer B’ containing 10 mM isoascorbic acid and 0.1% (v/v) Triton X-100, and then desalted with Sephadex G25 PD-10 column, and equilibrated with 125 mM Tris-HCl (pH 9.6). The activity of NAD-cyMDH and NADP-cyME were measured via spectrophotometer in a final volume of 1 ml at 340 nm. NAD-cyMDH was assayed in the oxaloacetate reduction with 800 mM Tris-HCl (pH 8.2), medium containing 200 mM KHCO_3_, 40 mM MgCl_2_, 10 mM GSH and 3 mM NADH (Lara et al., 2004). The assay mixture of NADP-cyME contained 50 mM Tris-HCl (pH 7.4), 10 mM MgCl_2_, 0.5 mM NADP and 10 mM malate (Detarsio et al., 2003).

Phosphoenolpyruvate carboxylase (PEPC, EC 4.1.1.31) was extracted with 2 ml of 100 mM Tris-HCl buffer (pH 8.2) containing 1 mM EDTA, 7 mM 2-mercaptoethanol, 5% (v/v) glycerin, and 3% (w/v) PVPP. The homogenate was centrifuged at 4°C for 15 min at 18,000×***g***, and 1 ml of supernatant was adjusted to 40% ammonium sulfate, kept at 4°C for 2 h and centrifuged at 16,000×***g*** for 15 min. The reaction medium contained 50 mM Tris-HCl (pH 9.2), 4 mM PEP, 10 mM MgSO_4_, 10 mM NaHCO_3_, 0.1 mM NADH and 5 U malate dehydrogenase (Wang et al., 2010).

A Phosphoenolpyruvate carboxykinase (PEPCK, EC. 4.1.1.32) assay was performed as described by Walker et al. (1999) with some modification. The extraction medium contained 200 mM Bicine-KOH (pH 9.0) and 50 mM DTT. PEPCK activity was assayed in the carboxylase direction resulting in the oxidation of NADH at 340 nm with a reaction medium including 100 mM HEPES (pH 7.0), 100 mM KCl, 90 mM KHCO_3_, 5 mM PEP, 1 mM ADP, 10 mM MnCl_2_, 4% (v/v) 2-mercaptoethanol, 4 Mm MgCl_2_, 0.14 mM NADH, 6 U of malate dehydrogenase and enzyme extract (Muhaidat and McKown, 2013). One unit of PEPCK activity corresponds to the production of 1 μmol of product per minute. The sugar-metabolic and malate-metabolic enzymes were all measured from three biological replications.

### Data analysis

The data was analyzed using one-way analysis of variance (ANOVA). Statistical analyses were conducted using SAS software (SAS Institute, Cary, USA), and means were compared using Duncan's New Multiple Range Test at *P*≤0.05.
